# Supplementary material for: Regulation of Smooth Muscle Cell Proliferation by Mitochondrial Ca2+ in Type 2 Diabetes
Source: Int J Mol Sci. 2023 Aug 17;24(16):12897. doi: 10.3390/ijms241612897 (PMC10454141; doi:10.3390/ijms241612897)
Supplement: Supplementary file 1 [file ijms-24-12897-s001.zip › ijms-2522754-supplementary.pdf]

A

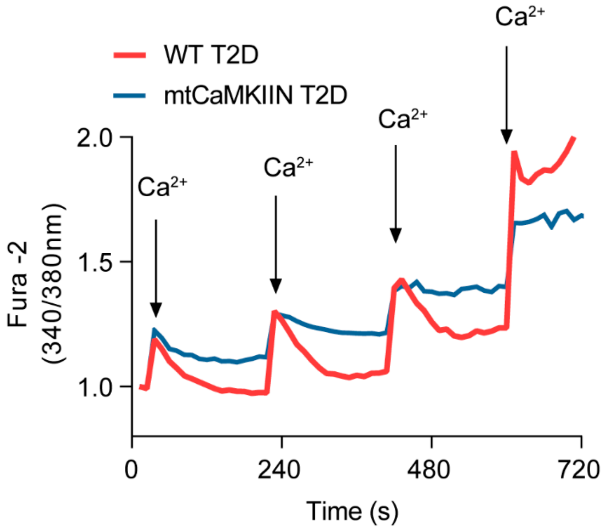

Figure S1. Mitochondrial Calcium uptake in permeabilized VSMC isolated from WT and mtCaMKIIN T2D mice assessed by Fura-2, in response to 0.5 μM $CaCl_2$.

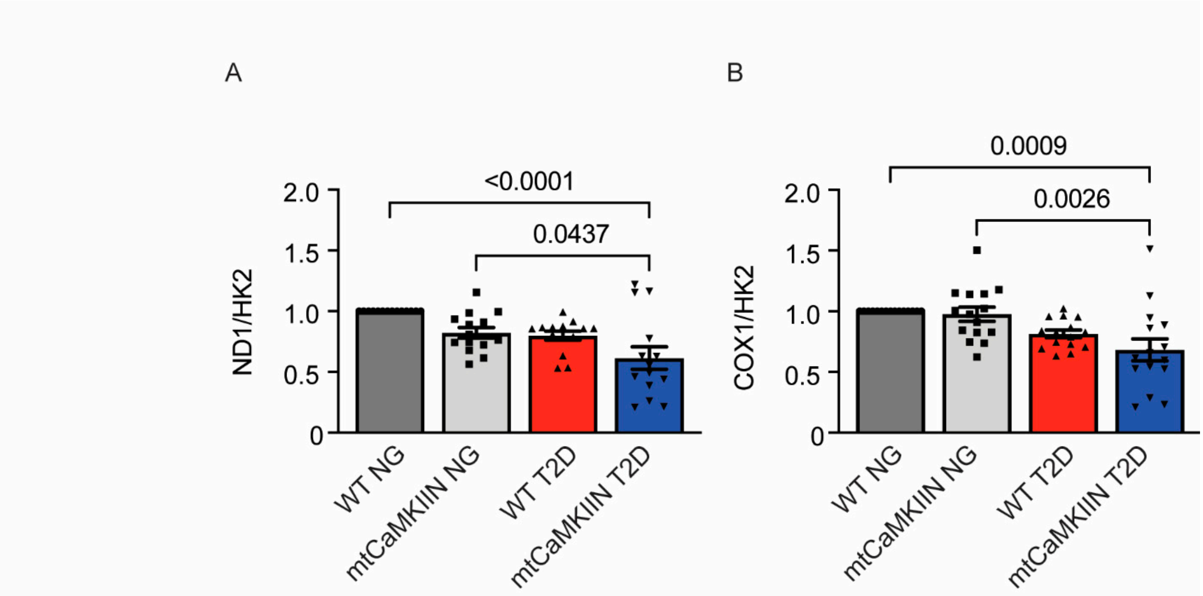

Figure S2. Mitochondrial DNA copy number in VSMCs from NG and T2D WT and mtCaMKIIN mice.
